# Cost-effectiveness of iStent-based trabecular micro-bypass stenting combined with cataract surgery in mild-to-moderate open-angle glaucoma: a systematic review of analytic perspectives and time horizons

**DOI:** 10.1186/s12962-026-00846-6

**Published:** 2026-10-20

**Authors:** Dan Drobin

**Affiliations:** Department of Clinical Neuroscience, Karolinska Institutet, Stockholm, Sweden

**Keywords:** Trabecular micro-bypass stent, Open-angle glaucoma, Cost-utility analysis, Analytic perspective, Time horizon

## Abstract

**Background:**

Glaucoma remains a major cause of irreversible blindness and was estimated to affect 64.3 million people worldwide in 2013. Despite growing clinical use of minimally invasive glaucoma surgery (MIGS), reimbursement and uptake remain constrained by uncertainty about long-term cost-effectiveness. iStent-based trabecular micro-bypass implantation is commonly performed at the time of cataract surgery in patients with mild-to-moderate open-angle glaucoma, yet its economic value has not been synthesised systematically across healthcare systems.

**Objective:**

To evaluate the cost-effectiveness of iStent trabecular micro-bypass implantation performed concurrently with cataract surgery for mild-to-moderate open-angle glaucoma, and to examine how the analytic perspective and time horizon adopted in the underlying models relate to the decision the evidence is intended to inform.

**Methods:**

A systematic review was performed of published model-based economic evaluations. Eligible studies used a formal decision-analytic model, reported quality-adjusted life years (QALYs), and evaluated an iStent device implanted concurrently with cataract surgery. PubMed, Embase, the Cochrane Library and Web of Science were searched on 27 July 2026 without date, language or publication-type restriction. Incremental cost-effectiveness ratios (ICERs), QALYs, analytic perspectives, time horizons, discount rates and probabilistic results were extracted with a page reference for each value. Reporting was appraised item-by-item against CHEERS 2022. Findings were synthesised narratively; no quantitative pooling was attempted. Reporting followed PRISMA 2020.

**Results:**

Of 418 records identified and 293 after de-duplication, eight economic evaluations from eight countries met the eligibility criteria. All eight compared the device added to cataract surgery against cataract surgery alone; seven used a cohort Markov model and one an individual-patient microsimulation. Six adopted a payer or health-system perspective and two a societal perspective; five used a lifetime horizon, one 35 years and two 15 years. No evaluation adopted a provider perspective or a base-case horizon shorter than 15 years. All eight reported a favourable base-case result in the mild-to-moderate target population: reported ratios included €1,154 per QALY in France, €12,676 in Spain, €13,037 in Italy, €10,955 to €15,673 in Germany, JPY 1,430,647 in Japan and A$2,430 in Australia, together with two instances of dominance. Six of the eight were funded by the device manufacturer. Incremental QALY gains were small throughout, between approximately 0.005 and 0.17, so the reported ratios are highly sensitive to small changes in the denominator. One study did not report a price year and for a second it could not be verified; no study reported a pre-specified health economic analysis plan, and none modelled long-term structural change in medication prices, prescribing practice or competing alternatives.

**Conclusions:**

The included peer-reviewed models generally project that adding an iStent-family device to cataract surgery can be cost-effective over long horizons. That projection depends on sustained medication reduction, on extrapolated glaucoma progression and on local device and treatment costs. Direct evidence that the modelled long-term clinical benefit is realised in practice and offsets the acquisition cost remains limited: the evidence base is small, concentrated in industry-funded analyses of a single device family drawing largely on the same two-year pivotal trial, and built on incremental QALY gains close to zero. All included evaluations adopt a payer, health-system or societal perspective over 15 years or longer, and none addresses whether the marginal cost of adding a stent to an already-scheduled cataract procedure is justified within an annual operating budget. This is a gap in the included QALY-based cost-utility literature; the review was not designed to identify provider-perspective budget-impact analyses outside these eligibility criteria.

**Trial registration:**

Not applicable. This study is a systematic review of published economicevaluations; no clinical trial registration was required. The review was not registeredprospectively in PROSPERO.

**Supplementary Information:**

The online version contains supplementary material available at https://doi.org/10.1186/s12962-026-00846-6.

## Key points

Eight model-based economic evaluations of iStent implanted concurrently with cataract surgery were identified across eight healthcare systems. All report a favourable base-case result in the mild-to-moderate target population, and six of the eight were funded by the device manufacturer. Every evaluation adopts a payer, health-system or societal perspective over 15 years or longer, and none models the provider perspective within an annual budget cycle. The device cost is incurred at the time of surgery, while the modelled savings are evaluated over 15 years or longer. Where the device is not separately reimbursed, the surgical service may bear that cost without recovering it within its own planning horizon, and adoption may require a decision-maker operating across a broader or more integrated budget. No included model reports the distribution of costs and savings between budget holders.

**Supplementary Information:**

The online version contains supplementary material available at https://doi.org/10.1186/s12962-026-00846-6.

## Introduction

Glaucoma is a leading cause of irreversible blindness worldwide, estimated to affect 64.3 million individuals in 2013 [1]; an earlier analysis had put the figure at 60.5 million in 2010 [2]. The disease is characterised by progressive optic nerve damage and visual field loss, most commonly associated with elevated intraocular pressure (IOP) [3]. Primary open-angle glaucoma (POAG) and primary angle-closure glaucoma (PACG) represent the two major clinical entities. The prevalence of POAG is highest in Africa, at approximately 4.20%, whereas the prevalence of PACG is highest in Asia, at approximately 1.09% [1]. The same analysis projected 76.0 million people affected in 2020 and 111.8 million in 2040, underscoring the escalating public health burden and the need for clinically effective and economically sustainable interventions [1].

The economic burden of glaucoma varies by disease stage and healthcare system. Direct costs include pharmacological treatment, surgical interventions and outpatient follow-up. Disease progression also reduces independence and health-related quality of life [3]. Long-term adherence to topical therapy is frequently poor due to side effects, complexity of treatment regimens, and financial constraints, contributing to inadequate IOP control and accelerated disease progression [3].

Cataract extraction can lower IOP, with a larger average effect in angle-closure than in open-angle disease, since lens removal deepens the anterior chamber and facilitates aqueous outflow [4]. A durable reduction has also been documented in ocular hypertension [5]. In open-angle disease, cataract surgery alone does not consistently eliminate the need for glaucoma treatment, prompting increasing interest in adjunctive surgical approaches such as minimally invasive glaucoma surgery (MIGS) [6].

The iStent platform (Glaukos Corp.) comprises a family of trabecular micro-bypass devices, including the original iStent, iStent inject^®^ and iStent inject^®^ W, designed for implantation at the time of cataract surgery. By bypassing the trabecular meshwork, these devices enhance physiological aqueous outflow and reduce IOP [6]. Evidence indicates that iStent-based implantation achieves greater and more sustained pressure reduction than cataract surgery alone [6] and is associated with reduced reliance on topical therapy, with potential downstream benefits for adherence, treatment persistence and long-term costs [6, 7].

Two features of this clinical situation shape the economic question and are central to the present review. First, when the stent is implanted concurrently with cataract surgery the marginal resource use is the device, a small amount of additional operating time and the associated consumables, incurred at the time of surgery; the procedure itself is already scheduled and funded. When the same device is implanted as a standalone procedure the marginal cost is the entire episode, including operating theatre, staff and follow-up. These are different decision problems and the resulting incremental cost-effectiveness ratios are not commensurable quantities. The present review is restricted to the concurrent situation.

Second, the device cost is incurred at the time of surgery. Where it is not separately reimbursed, the surgical provider may bear that cost, whereas the modelled savings — principally reduced topical medication use and deferred subsequent surgery — accrue over a longer period and may fall in other budgets. Whether an economic evaluation can inform a given adoption decision therefore depends on the analytic perspective and time horizon it adopts, and not only on the magnitude of the ratio it reports. This review consequently extracts and compares perspective and horizon as primary study characteristics alongside the reported ICERs.

## Materials and methods

This systematic review was reported in accordance with the PRISMA 2020 statement [8], and the reporting quality of the included economic evaluations was appraised against the CHEERS 2022 checklist [9]. The review was not registered prospectively in PROSPERO.

### Information sources and search strategy

PubMed, Embase (Elsevier), the Cochrane Library (Wiley) and Web of Science Core Collection (Clarivate) were searched on 27 July 2026. No date, language or publication-type restriction was applied at the search stage. The strategy combined a device block with a health-economics block; eligibility with respect to indication, model type and outcome measure was assessed at screening rather than imposed in the search, in accordance with Cochrane Handbook guidance on sensitivity in searching and specificity in screening.

A broader systematic review of decision-analytic models in primary open-angle glaucoma has been published [10]. It covers economic evaluations across the whole of primary open-angle glaucoma rather than a single device class, and does not address iStent implantation performed concurrently with cataract surgery as a separate question; the present review is narrower and complementary.

The verbatim strategy for each database, together with the platform, the search date and the number of records retrieved, is provided in Supplementary Table S1. Syntax differences between platforms are documented there, including the use of proximity operators in the Cochrane Library where truncation inside a quoted phrase is not supported, and the absence of a controlled vocabulary in Web of Science.

The searches retrieved 418 records: 68 from PubMed, 275 from Embase, 16 from the Cochrane Library (all from CENTRAL) and 59 from Web of Science. After de-duplication 293 unique records remained. Records were de-duplicated on digital object identifier, PubMed identifier and normalised title with year; five remaining candidate pairs above a fuzzy-matching threshold were resolved manually and are documented in Supplementary Table S1.

### Eligibility criteria

Studies were eligible if they met all of the following criteria.

Population. Adults with mild-to-moderate open-angle glaucoma. The population of each study is reported in Table 1. Angle-closure glaucoma and ocular hypertension without established glaucomatous damage were excluded.

**Table 1:** Characteristics of the eight included economic evaluations

Study	Country	Population	Device	Perspective	Time horizon	Model	Comparator	Funding
Ahmed 2020 [7]	Canada	OAG with cataract; 83.1% mild, 16.9% moderate	iStent inject (2 stents)	Public payer	15 years	Markov, HPA stages, monthly cycles	Cataract surgery alone	Glaukos, via Pharmerit (employees as co-authors)
Bartelt-Hofer 2020 [14]	Germany	POAG, stratified by stage	iStent, 1 and 2 stents	Payer	Lifetime, yearly cycles	Markov	Cataract surgery alone	None declared
Fea 2021 [15]	Italy	Mild-to-moderate OAG	iStent inject	Italian NHS	Lifetime	Markov, 4 health states, monthly cycles	Cataract surgery alone	Glaukos, via EBMA Consulting (employee as co-author)
Nieland 2021 [16]	France	Mild-to-moderate OAG	iStent inject	Societal	Lifetime	Markov, monthly cycles	Cataract surgery alone	Glaukos, via Pharmerit (employees as co-authors)
Teus 2021 [17]	Spain	Mild-to-moderate POAG	iStent inject	Health system	Lifetime, monthly cycles	Markov	Cataract surgery alone	Glaukos (employees as co-authors)
Healey 2022 [20]	Australia	OAG	iStent and iStent inject, pooled	Health system / third-party payer	15 years, deliberately limited	Individual-patient Monte Carlo microsimulation, 500,000 patients	Usual care: cataract surgery alone	Glaukos, via THEMA Consulting
Igarashi 2022 [18]	Japan	Mild-to-moderate POAG	iStent inject	Public payer	Lifetime, monthly cycles	Markov	Cataract surgery alone	Glaukos, via Creativ-Ceutical (employees as co-authors)
Sood 2022 [19]	USA	Mild-to-moderate POAG, age 65+	iStent inject	Societal; Medicare rates	Lifetime, 35 years	Markov, 4 health states	Cataract surgery alone	Research to Prevent Blindness

HPA = Hodapp-Parrish-Anderson; NHS = National Health Service; HTA = health technology assessment; OAG = open-angle glaucoma; POAG = primary open-angle glaucoma. Studies are listed chronologically. All eight evaluate an iStent device implanted concurrently with cataract surgery and all eight use cataract surgery alone, or usual care in which the cataract procedure is performed without a stent, as the comparator

Full text of Sood et al. was not accessible to the author’s institution; that study was appraised from the published abstract and the publisher’s article preview, which state the funding source and the absence of proprietary or commercial interest. See Sect.  4.1

Intervention. An iStent or iStent inject^®^ trabecular micro-bypass stent, implanted through the trabecular meshwork into Schlemm’s canal. Other minimally invasive glaucoma surgery devices were excluded on anatomical and mechanistic grounds: the Hydrus Microstent dilates Schlemm’s canal rather than creating a point bypass; XEN and PreserFlo drain to the subconjunctival space; and OMNI, Kahook Dual Blade, GATT and Trabectome perform canaloplasty or trabeculotomy without implanting a stent. Studies in which an iStent device appeared only as a comparator were excluded, since the reported ratio then applies to the other device.

Concurrent cataract surgery. The stent had to be implanted at the time of cataract surgery. Standalone implantation was excluded for the reason set out in the Introduction. This criterion was specified after the review was begun rather than at the outset, and is reported as a deviation from the original plan.

Model type. A formal decision-analytic model. Both cohort Markov models and individual-patient microsimulations were eligible. Model type is reported as a study characteristic in Table 1 and discussed as a source of heterogeneity in Sect.  4.1. Costing studies without a formal model were excluded.

Outcome measure. Quality-adjusted life years. Cost-minimisation analyses and cost-effectiveness analyses using other natural units, such as progression-free life years, were excluded, since ratios expressed in different units cannot be compared against a common willingness-to-pay threshold.

Publication type. Primary peer-reviewed economic evaluations. Health technology assessment reports, conference abstracts, correspondence, editorials, registry entries and narrative reviews were excluded.

### Study selection and data extraction

**Fig. 1:**
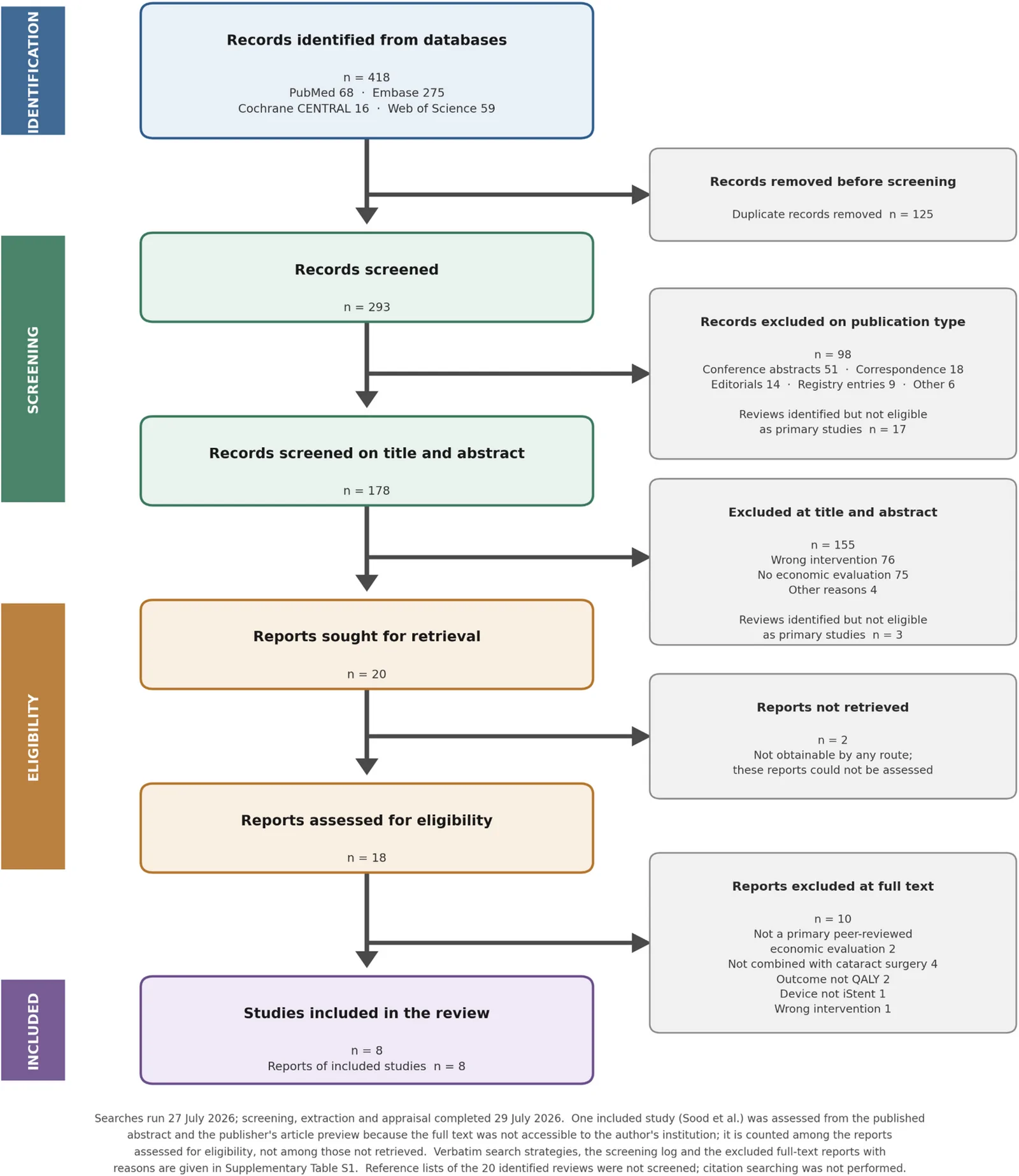
PRISMA 2020 flow diagram of study selection. QALY = quality-adjusted life year. Verbatim search strategies for each database, the documented syntax deviations between platforms,the screening log and the list of excluded full-text reports with reasons are provided in Supplementary Table S1

Records were first filtered mechanically on publication type; 98 records were excluded at this stage and 17 reviews were identified but not eligible as primary studies. The remaining 178 records were screened on title and abstract by the author; 155 were excluded and a further three reviews were identified but not eligible as primary studies. Twenty reports were sought for full-text assessment, of which two could not be retrieved. Ten reports were excluded at full text and eight studies were included. The full selection process is shown in Fig. 1, and every excluded full-text report is listed with its reason for exclusion in Supplementary Table S1. Reference lists of the 20 reviews identified were not screened; citation searching was not performed.

Searches were run on 27 July 2026. Screening, full-text assessment, data extraction and appraisal were carried out by a single reviewer (DD). No independent duplicate screening was undertaken and no inter-rater agreement could therefore be calculated; this is stated as a limitation in Sect.  4.1. To mitigate the risk of transcription error, every extracted value was recorded with a page or section reference to the source document, and the complete extraction table with these references is provided as Supplementary Table S1.

Extracted variables follow the parameters that define a cost-effectiveness analysis in standard methodological guidance [11]: country, analytic perspective, time horizon, model type and cycle length, comparator, device modelled, currency, price year, discount rates for costs and outcomes, incremental costs and QALYs, the incremental cost-effectiveness ratio, the willingness-to-pay threshold applied in the source study, the reported probability of cost-effectiveness at that threshold, the funding source and declared conflicts of interest.

### Appraisal of reporting

Each included study was appraised item-by-item against the 28 items of CHEERS 2022 [9]; study-level results are presented in Supplementary Table S2 and summarised in Sect. 3.1. CHEERS is a reporting standard and not a risk-of-bias instrument. No generally accepted, fully validated risk-of-bias instrument is in widespread use for model-based economic evaluations; the ECOBIAS checklist has been developed for this purpose but is not yet fully validated or widely adopted [12]. Reporting completeness is therefore interpreted here as a measure of methodological transparency and not as an estimate of internal validity. Because each item asks whether a given piece of information is reported, the appraisal is largely a factual check rather than a graded judgement, which is the basis on which it was undertaken by a single reviewer. Several items nonetheless require interpretation, and single-reviewer appraisal remains a limitation.

The reporting of parameter uncertainty was additionally considered against the ISPOR-SMDM Modeling Good Research Practices recommendations on parameter estimation and uncertainty [13], as a narrative check rather than a scored assessment. No formal risk-of-bias grading was performed and none is reported.

Given the heterogeneity across healthcare systems, currencies, price years and model structures, findings were synthesised narratively. No quantitative pooling was attempted, and ICERs were not converted to a common purchasing-power-adjusted currency because price years were not consistently reported.

### Use of AI tools

Claude (Anthropic) was used with author supervision at several stages of this review. The author designed the conceptual search blocks; the tool was used to translate them into the syntax of each database platform, and the author verified every platform translation and ran the searches. The tool was used to parse the exported records and to propose de-duplication; the author reviewed every de-duplication decision. The tool was used to extract data from the retrieved full texts into the structured table provided as Supplementary Table S1; every extracted value carries a page or section reference and was verified against the source document by the author. The tool was used to draft manuscript text, which the author revised and approved. Every eligibility decision was made by the author. No AI tool is credited as an author, and the author assumes full responsibility for the content of the manuscript and all supplementary material.

## Results

### Study characteristics

Eight economic evaluations met the eligibility criteria. The study selection process is illustrated in Fig. 1 and the characteristics of the included studies are summarised in Table 1.

The studies were conducted in eight countries: Canada [7], Germany [14], Italy [15], France [16], Spain [17], Japan [18], the United States [19] and Australia [20]. All eight modelled an iStent device implanted concurrently with cataract surgery, and all eight used cataract surgery alone as the comparator.

The included population is defined as mild-to-moderate disease throughout, but only one evaluation reports results by disease stage. Bartelt-Hofer and Flessa report moderate and advanced primary open-angle glaucoma separately; no evaluation reports mild disease separately from moderate. Ahmed et al. state a cohort of 83.1% mild and 16.9% moderate disease but report a pooled result.

Four evaluations specify primary open-angle glaucoma and four state open-angle glaucoma without further qualification. None models secondary open-angle glaucoma separately, so the results cannot be read as applying to pseudoexfoliative or pigmentary glaucoma.

Six evaluations modelled iStent inject exclusively. One (Bartelt-Hofer and Flessa) modelled first-generation iStent with one-stent and two-stent strategies as separate arms, and one (Healey et al.) pooled the two generations, treating them as a single trabecular bypass device class. A subgroup analysis by generation is not feasible on this distribution, and residual heterogeneity from device generation cannot be excluded.

Seven evaluations used a cohort Markov model, with cycle lengths reported as monthly in five studies and yearly in one and not stated in one. The eighth (Healey et al.) used an individual-patient Monte Carlo microsimulation of 500,000 simulated patients. Both are decision-analytic models and both meet the eligibility criteria; the difference is noted because microsimulation permits patient-level heterogeneity that a cohort model cannot represent, while in this instance it also precluded probabilistic sensitivity analysis on grounds of computational burden.

The analytic perspective was that of a public payer or health system in six studies and societal in two (Nieland et al., France; Sood et al., United States), although Sood et al. cost resources at Medicare rates. No study adopted a provider or hospital-budget perspective.

Time horizons were lifetime in five studies, 35 years in one (Sood et al.) and 15 years in two (Ahmed et al.; Healey et al.). Healey et al. state that the horizon was deliberately limited to 15 years to avoid extrapolating beyond the available evidence. No evaluation modelled a base-case horizon shorter than 15 years, although Ahmed et al. report a five-year scenario analysis, considered further in Sect.  3.2.

Six of the eight evaluations disclose a relationship with the device manufacturer. All six were funded by Glaukos Corporation. In five (Ahmed et al., Fea et al., Nieland et al., Teus et al. and Igarashi et al.) one or more co-authors were employees of the manufacturer, and in at least five the analysis was carried out by a health-economics consultancy funded by the manufacturer. One (Bartelt-Hofer and Flessa) declares no specific funding and no conflict of interest. One (Sood et al.) reports support from Research to Prevent Blindness with no proprietary or commercial interest declared. This concentration is considered further in Sect.  4.

Reporting quality against CHEERS 2022 is presented for each study in Supplementary Table S2. Reporting was substantially complete for the study question, comparators, perspective, model description and headline results in all studies. Two items were not met by any study: no evaluation reported a pre-specified health economic analysis plan (item 4), and none characterised distributional effects (item 19). Patient or stakeholder engagement (items 21 and 25) was reported by none of the eight. The price year was reported in six of eight studies and not reported in one (Fea et al.); for the eighth (Sood et al.) it could not be verified because the full text was not accessible (item 15). Where the price year is absent the reported costs cannot be adjusted to a common year.

### Economic outcomes

The reported result of each study, the threshold applied in that study and the relationship between the two are set out in Table 2.

**Table 2:** Reported result of each study in relation to the willingness-to-pay threshold applied in that study

Country [ref]	Reported result (original currency)	Threshold applied in source study	Formally endorsed national threshold	Result relative to that threshold
Canada [7] Ahmed 2020	Dominant. ICUR − CAD 16,691; incremental cost − CAD 389.00 (95% CI − 1,712.00 to + 850.70); +0.023 QALY (95% CI 0.004–0.044); 95% of simulations below CAD 62,366	CAD 50,000 and CAD 100,000 per QALY	No	94.1% of simulations below CAD 50,000; 97.0% below CAD 100,000; 73.7% probability of cost saving
Germany [14] Bartelt-Hofer 2020	Synthesis arm, moderate POAG: €15,673.41 per QALY (one stent); €10,955.20 per QALY (two stents). Advanced POAG, outside the mild-to-moderate target population and reported for context: €86,942.73 and €60,269.14 per QALY	1 × GDP per capita, €43,433.87 per QALY	No	Both moderate-stage strategies well below the threshold; both advanced-stage strategies above it. The source reports that more than 95% of simulations in the overall probabilistic analysis fell below the threshold; arm- and stage-specific probabilities were not reported
Italy [15] Fea 2021	ICER €13,037 per QALY	Conventional Italian range €25,000–50,000 per QALY	No	Approximately 26–52% of that range; 98% probability of cost-effectiveness at €50,000
France [16] Nieland 2021	ICER €1,154 per QALY	€30,000 per QALY (conventional)	No	Approximately 4% of that threshold; 86% probability of cost-effectiveness
Spain [17] Teus 2021	ICER €12,676.48 per QALY (lifetime base case); +0.083 QALY (12.36 vs. 12.27); incremental cost €1,002.07 as reported, see footnote 4. Scenario analysis at 15 years: €15,699.56 per QALY	€21,000 and €30,000 per QALY	No	Approximately 60% and 42% of those thresholds respectively; 78.4% and 90.9% probability of cost-effectiveness
Australia [20] Healey 2022	A$2,430 per QALY in the cataract surgery population; incremental cost A$177; +0.0726 QALY. Standalone population, not in the synthesis: A$14,644 per QALY	A$50,000 per QALY (conventional)	No	Approximately 5% of that threshold. No probabilistic analysis was performed, on grounds of computational burden
Japan [18] Igarashi 2022	ICUR JPY 1,430,647 per QALY (base case, lifetime horizon); total cost JPY 1,025,785 vs. 933,759; 12.80 vs. 12.74 QALY (+ 0.06)	JPY 5,000,000 per QALY	Yes — Chuikyo/C2H; governs reimbursement price adjustment rather than coverage	Approximately 29% of that threshold; 90% probability of cost-effectiveness
USA [19] Sood 2022	Dominant. USD 49,599.86 and 12.21 QALY versus USD 54,409.25 and 12.04 QALY for cataract surgery alone: USD 4,809.39 less and + 0.17 QALY	USD 50,000 per QALY	No	Dominant; 94.19% of iterations cost-effective at that threshold

ICER = incremental cost-effectiveness ratio; ICUR = incremental cost-utility ratio; QALY = quality-adjusted life year; CI = confidence interval; GDP = gross domestic product; POAG = primary open-angle glaucoma

Footnote 1. Among the thresholds applied in the included evaluations, the Japanese value (JPY 5,000,000 per QALY, set by Chuikyo and administered by C2H) was the only formally specified national price-adjustment threshold, and it governs reimbursement price adjustment rather than coverage. The values shown for the other countries are widely cited conventional reference ranges rather than decision rules, so the percentages are indicative only

Footnote 2. ICERs have not been converted to a common purchasing-power-adjusted currency. Price years, where reported, span 2017 to 2021; one study does not report one and for a second it could not be verified. Conversion to a common year is therefore not possible for the whole set. See Sect.  4.1

Footnote 3. Incremental QALY gains are small throughout, from approximately 0.005 to 0.17. The ratios are correspondingly sensitive to small changes in the denominator and should not be compared between studies as though they were stable quantities. See Sect.  3.2

Footnote 4. The results table in Teus et al. reports an incremental cost of €1,002.07, while the two total costs shown in the same table differ by €1,050.19; the reported ICER is consistent with the latter. The figure as reported is given here. That table also carries a footnote reading “Time horizon 10 years”; the methods section states a lifetime horizon and identifies ten years as the period over which treatment efficacy was extrapolated, after which the model continues to end of life without further effect

All eight evaluations reported results favourable to the intervention in the mild-to-moderate target population. The reported ratios were €1,154 per QALY in France, €10,955 for two stents and €15,673 for one in Germany, €12,676 in Spain, €13,037 in Italy, JPY 1,430,647 in Japan and A$2,430 in Australia, the last from an incremental cost of A$177 and a gain of 0.0726 QALYs. Two analyses reported dominance, that is lower cost with greater effect: Ahmed et al. report an incremental cost-utility ratio of − CAD 16,691 with a 73.7% probability of cost saving and a 94.1% probability of falling below CAD 50,000 per QALY, and Sood et al. report the intervention costing USD 4,809.39 less and yielding 0.17 more QALYs than cataract surgery alone, with 94.19% of iterations cost-effective at USD 50,000 per QALY.

A feature common to the whole evidence base deserves emphasis. The incremental QALY gains reported are small: approximately 0.005 to 0.013 in Germany depending on the number of stents, 0.023 in Canada (Ahmed et al., 95% confidence interval 0.004 to 0.044), 0.06 in Japan, 0.0726 in Australia, 0.083 in Spain and 0.17 in the United States, the largest gain in the material. Because the ratio is a quotient with this quantity in the denominator, small differences in modelled effectiveness produce very large differences in the reported ICER. The German analysis illustrates this directly: the same procedure yields €10,955 per QALY in moderate disease and €60,269 per QALY in advanced disease, where the utility gain is smaller while the cost is unchanged. The reported ratios should therefore not be treated as stable quantities suitable for comparison between studies.

Time horizon has a demonstrable effect on the conclusion. Ahmed et al. report the same model over several horizons: the intervention is dominant over a lifetime and over the 15-year base case, but at five years the cost-saving result does not persist: the incremental cost-utility ratio is CAD 32,227.80 per QALY with an incremental cost of CAD 446.90. That ratio remains below the CAD 50,000 threshold applied in that study, so the intervention is still reported as cost-effective at five years, but the uncertainty is substantially wider, with a 95th percentile of CAD 125,941 against CAD 55,952 over a lifetime. The reason is visible in the cost breakdown: the additional primary surgery cost of CAD 1,671.50 falls in the first year, whereas the medication saving of CAD 2,069.60 accumulates across the whole horizon.

Teus et al. report the same pattern from a second model. Their lifetime base case gives €12,676.48 per QALY; a scenario analysis restricted to 15 years gives €15,699.56. The direction is the same as in Ahmed et al.: a shorter horizon yields a less favourable ratio, because less of the projected saving has accumulated.

A third evaluation points the other way. Sood et al., one of the two evaluations without manufacturer funding, state in their abstract that the device arms “dominated or were cost-effective compared with cataract surgery alone within 5 years”. The full text was not accessible (Sect.  4.1), so the underlying five-year figures and the way they were derived could not be assessed, and this result is not directly comparable with the scenario analyses of Ahmed et al. and Teus et al.

Probabilistic results were reported inconsistently. Where a numerical probability of cost-effectiveness at a threshold applied in the source study was reported, the values were 98% in Italy, 94.19% in the United States, 94.1% in Canada (Ahmed et al.), over 95% in Germany, 90.9% in Spain at a threshold of €30,000 and 78.4% at €21,000, 90% in Japan and 86% in France. One evaluation reported none: Healey et al. state that probabilistic sensitivity analysis was not performed because of the computational burden of the microsimulation. These figures should not be read as a pooled probability, because the underlying thresholds, model structures, perspectives and costing frameworks differ between studies.

### Clinical effectiveness and shared source data

The included studies reported superior clinical outcomes for iStent combined with cataract surgery compared with cataract surgery alone. Reductions in intraocular pressure were consistently greater in the iStent arms, and medication burden was reduced.

Six of the eight evaluations modelled iStent inject, and their clinical-effect inputs were derived largely from the same two-year pivotal trial [6]. The apparent convergence across the economic models therefore does not represent independent replication. Extrapolation beyond the two-year trial follow-up rests on assumption in every model. Three evaluations state the assumption explicitly. Ahmed et al. assume that the treatment effect declines by 5% a year from the end of year three to the end of year ten, with no effect thereafter, and Igarashi et al. apply the same 5% annual decline with no effect after ten years. Both describe this as conservative, in that the effect is assumed to be lost faster than the data they cite suggest. Teus et al. extrapolate treatment efficacy for ten years, after which the model continues to end of life without further effect. Nieland et al. state only that efficacy was extrapolated over a lifetime from the two-year trial results, and for the remaining four no explicit projection method was identified, so how much of each model’s benefit rests on assumption rather than on trial data cannot be compared between studies.

## Discussion

All eight included peer-reviewed model-based evaluations reported a favourable base-case result, although four features of the evidence base qualify that finding considerably.

The first is the size and composition of the evidence base. Eight evaluations of a single device family, six of them funded by the manufacturer and six drawing their clinical-effect inputs largely from the same two-year pivotal trial, provide less independent information than the count suggests (Sect.  3.3).

No difference in the direction of the reported results between industry-funded and independent evaluations was apparent. Two of the eight have no manufacturer relationship — Bartelt-Hofer and Flessa declare no specific funding and no conflict of interest, and Sood et al. report support from a non-commercial foundation — and both report results in the same direction as the six industry-funded evaluations. Since every included evaluation reports a favourable base-case result, no formal comparison between the two groups is possible, and this material can neither demonstrate nor exclude an effect of funding source on the reported ratios. The concentration itself, six of eight, remains a limitation of the evidence base.

The second is the magnitude of the modelled benefit. Incremental QALY gains between approximately 0.005 and 0.17 correspond to a range from negligible to approximately two months of full-health-equivalent time over a lifetime. Ratios computed from such small denominators are unstable, and the German analysis demonstrates a nearly sixfold change in the reported ratio for the same procedure performed at a different disease stage. This does not indicate an error in the source studies; it is a property of the decision problem, and it argues against treating the ratios as directly comparable.

The third concerns what the models do not represent. Among the seven evaluations available in full text, discount rates were constant and, where a numerical rate was stated, ranged from 1.5% to 5%; the discount rate could not be verified for Sood et al. Seven of the eight characterise parameter uncertainty probabilistically in the manner current modelling guidance recommends [13]. The review identified no explicit modelling of long-term structural change in medication prices, prescribing practice or competing treatment alternatives.

In the only model providing a detailed cost breakdown, the net saving arose primarily from reduced topical medication use (Sect.  3.2). The included models project such savings over horizons of fifteen years or longer. Over such a period the price of topical prostaglandins, the prescribing regimen and the available alternatives may all change, and any of these changes could materially alter the projected saving. A probabilistic analysis that varies the current price within its confidence interval does not represent that possibility, because it holds the structure fixed.

Discounting does not represent it either. The social discount rates applied in these models express time preference; they do not carry a project-specific risk premium, and the conventional position is that project risk should be represented in the model rather than in the rate. In this material it is represented in neither. The weighting is not trivial: for a constant annual saving over fifteen years, most of the present value falls after year five at every discount rate applied in the included models, from 1.5% to 5%, and year five is already beyond any observed follow-up.

Broader public-sector appraisal guidance offers an analogy for handling systematic optimism in long-term projections: United Kingdom central government appraisal requires an explicit adjustment that increases estimated costs and decreases and delays estimated benefits, on the evidence that appraisals systematically overstate benefits and understate costs [21]. Such adjustments are not a standard requirement for health-technology cost-utility models. No included evaluation applies one, and the projected saving enters each model at its full modelled value for the whole of the horizon.

This is an observation about the included evaluations rather than about the methods available. Value-of-information analysis could inform whether adoption should proceed under current uncertainty or await further evidence, and none of the included evaluations applies it.

Two evaluations report results from their own model over more than one horizon, and both show the conclusion to be horizon-dependent in the same direction. Ahmed et al. lose the cost-saving result at five years, and Teus et al. report a less favourable ratio at 15 years than over a lifetime (Sect.  3.2). Both are industry-funded. The independent evaluation by Sood et al. reports in its abstract that the intervention was dominant or cost-effective within five years, but the underlying figures could not be assessed (Sect.  3.2).

Loss of treatment effect over time is not specific to the intervention. Open-angle glaucoma is chronic and progressive, and in the included models the comparator arm also progresses and escalates to further treatment as the disease advances. The economic result depends on the difference between the arms, and therefore on how long the additional pressure reduction from the stent is assumed to persist, not on the absolute decline in either arm. The horizon dependence described above arises mainly from timing: the device cost falls in the year of surgery, while the medication saving accumulates year by year, so a shorter horizon captures less of it. Where the effect is assumed to wane, as in Ahmed et al. and Igarashi et al., the modelled clinical benefit is concentrated in the early years after surgery, which is also the period closest to the available trial data.

The fourth concerns the analytic perspective, and is the observation this review was extended to examine. Every included evaluation adopts a payer, health-system or societal perspective, and every base-case horizon is 15 years or longer. Not one adopts the perspective of the provider that purchases the device. The consequence is set out in Sect.  4.2.

### Strengths and limitations

The principal strength of this review is that eligibility was specified at device level and at the level of the decision problem, so that the included evaluations address a single, well-defined question: whether adding an iStent to a cataract operation that is already indicated represents value for money. Restricting inclusion in this way removes the heterogeneity in comparator structure that would otherwise dominate any comparison. A further strength is that every extracted value carries a page or section reference to its source where the full text was available, so the extraction is auditable.

Several limitations should be acknowledged.

The evidence base is small and concentrated. Six of the eight evaluations were funded by or co-authored with the device manufacturer, which limits confidence in the independence of the evidence base.

Model architecture differs across the included studies. Seven are cohort Markov models and one is an individual-patient microsimulation, which handle patient heterogeneity differently, so results are not strictly commensurable on that account.

Screening, full-text assessment, data extraction and appraisal were performed by a single reviewer, so no inter-rater agreement could be calculated and screening error cannot be excluded. The review was not registered prospectively in PROSPERO, and the eligibility criterion requiring concurrent cataract surgery was specified after the review was begun rather than at the outset.

Quality appraisal used CHEERS 2022, which measures reporting completeness rather than risk of bias (Sect.  2.4), so the internal validity of the individual models was not graded formally. Because reference lists were not screened and no citation searching was performed (Sect.  2.3), eligible studies not indexed in the four databases may have been missed.

One included study (Sood et al.) could not be obtained in full text because the author’s institution does not subscribe to the journal. It was assessed from the published abstract and the publisher’s article preview, which together establish the model class, time horizon and comparator; eight CHEERS items could not be assessed for that study. Two further records could not be retrieved despite multiple routes and remain unassessed; both are reported as such in Fig. 1.

ICERs are reported in the original currencies and were not converted to a common purchasing-power-adjusted currency. Price years span 2017 to 2021 where reported, are absent in one study and could not be verified in a second, so conversion to a common year is not possible for the whole set and would create an impression of comparability the underlying data do not support.

The exclusion of health technology assessment reports as a publication type removed from the synthesis a government-commissioned assessment that contains an original economic model and reports a less favourable ratio than any included study. The criterion is the one stated in the eligibility criteria for this review and was not altered, but the consequence should be stated: the included set is confined to peer-reviewed primary evaluations, and the uniformly favourable direction of their results should be read in that light.

No included evaluation reports results separately for mild and for moderate disease, although the population is defined as mild-to-moderate throughout. The reported ratios are therefore averages over a mixed severity distribution, and that distribution differs between studies where it is stated at all. This matters because, in the one evaluation providing a detailed cost breakdown, the modelled net saving arose almost entirely from reduced topical medication use, and medication burden differs by disease stage. The one evaluation that does stratify shows a steep gradient across stages: Bartelt-Hofer and Flessa report €10,955 per QALY for two stents in moderate disease against €60,269 for the same strategy in advanced disease. Whether a comparable gradient exists between mild and moderate disease is not established by any study identified here.

One inconsistency in a source publication should be recorded because it bears directly on the horizon comparison. The results table in Teus et al. carries a footnote reading “Time horizon 10 years”, whereas the methods section and abstract of the same paper state a lifetime horizon; ten years is the period over which treatment efficacy was extrapolated, after which the model continues to end of life without further effect. The accumulated QALYs reported, 12.36 and 12.27, are not attainable over ten years and confirm the lifetime reading. The study is classified here on its stated analytic horizon.

The geographic composition of the evidence base is narrow. All eight included evaluations derive from high-income health systems in Western Europe, North America, Japan and Australia. Device acquisition costs, physician fees, facility costs and the opportunity cost of cataract surgery capacity differ substantially between these settings and lower-resource ones, and the direction of those differences is not uniform: unit device prices may be higher while staff costs are lower. Generalisation of these findings to middle- or low-income health systems is not supported by the present evidence base.

Finally, as with all decision-analytic modelling, the reported results depend on assumptions about disease progression, utilities and cost inputs, and on extrapolation well beyond the two-year trial evidence available.

### Implications for clinical practice and policy

For the practising clinician the findings can be summarised as follows. The evidence applies to adults with mild-to-moderate primary open-angle glaucoma who are already scheduled for cataract surgery, in high-income health systems. It does not address secondary open-angle glaucoma such as pseudoexfoliative or pigmentary glaucoma, angle-closure disease or standalone implantation. The one evaluation that reports advanced disease separately finds a much less favourable ratio at that stage, and no evaluation distinguishes mild from moderate disease. The modelled health gain is small and arises from slower progression, while the modelled saving arises mainly from reduced medication use. Most of both is projected beyond the two years for which trial data exist. Within that population the included models consistently project an acceptable cost per QALY from the payer or health-system perspective, but they do not show that the benefit is realised in routine practice, and they do not inform decisions for patients outside that population.

The included economic models incorporate greater intraocular pressure reduction and lower medication burden for iStent combined with cataract surgery than for cataract surgery alone. These clinical inputs derive largely from the randomised evidence base for iStent inject, which rests on a single two-year trial, and were not independently re-evaluated in this review, which appraised economic models rather than clinical effectiveness. The modelled reduction in medication burden is nonetheless of practical relevance in routine care, given the well-documented difficulties of long-term adherence to topical therapy.

From a policy perspective, the more important observation is structural rather than numerical. The marginal resource use of adding a stent to a scheduled cataract operation is the device, some additional operating time and the associated consumables, and that cost is incurred in the year of surgery. The modelled benefit — reduced topical medication use and deferred subsequent surgery — is evaluated over horizons that every included model puts at 15 years or longer. In Ahmed et al., cost saving did not persist at five years, although the intervention remained cost-effective at the threshold used in that study.

The proportional weight of the additional operating time will differ between providers according to their baseline theatre throughput, but no included evaluation was identified as explicitly varying operating-time costs according to provider throughput. The magnitude of the effect is not established by the present review.

The practical consequence depends on how the provider is funded. Where a surgical service operates on an annual budget, where the device is not separately reimbursed within the procedure tariff, and where the topical medication whose reduction generates most of the saving in the one model providing a detailed cost breakdown is carried on a different budget entirely, the marginal cost falls wholly on the service in the year of surgery while none of the modelled return accrues to it within its planning horizon. Under those conditions the investment may not be recovered at provider level, and this follows from the funding structure rather than from any uncertainty in the underlying models.

Two evaluations label their base-case perspective societal. The non-health-sector cost components were, however, limited or incompletely reported, and the models do not establish that productivity or informal-care savings constitute a material share of the projected return.

Under these conditions adoption may require a decision-maker operating across a broader or more integrated budget than the surgical service, whose horizon and scope match the period and the sectors over which the modelled benefit accrues. The included models do not provide what such a decision-maker needs, because none of them reports the position of the provider or the distribution of costs and savings between budget holders.

This is a gap in the included QALY-based cost-utility literature. The review was not designed to identify provider-perspective budget-impact analyses outside these eligibility criteria. It identifies a concrete direction for future work: a budget-impact analysis from the provider perspective over a one to three year horizon, reporting explicitly which budget carries each cost and each saving, would address a decision that the included cost-utility analyses do not.

## Conclusions

The included peer-reviewed models generally project that adding an iStent-family trabecular micro-bypass device to already planned cataract surgery can be cost-effective over long horizons. All eight included peer-reviewed analyses across eight high-income healthcare systems report a favourable ratio or outright dominance in the mild-to-moderate target population. Six of the eight were funded by the device manufacturer, and six drew their clinical-effect inputs largely from the same two-year pivotal trial, so the consistency of direction is not independent replication.

That projection is conditional. It depends on sustained reduction in topical medication use, on extrapolated glaucoma progression beyond the two-year trial evidence available, and on local device and treatment costs. None of the included models explicitly represented long-term structural change in medication prices, prescribing practice or competing alternatives, and at the discount rates applied the greater part of the projected saving falls beyond any observed follow-up. Direct evidence that the modelled long-term clinical benefit is realised in practice and offsets the acquisition cost remains limited, and the distinction between modelled long-term cost-effectiveness and demonstrated long-term value should be preserved when these findings are used.

The evidence base is small, concentrated in analyses affiliated with the device manufacturer, and built on incremental QALY gains between approximately 0.005 and 0.17. The conclusion therefore rests on directional consistency rather than on a pooled quantitative estimate, and the reported ratios should not be compared between studies as though they were stable quantities.

Every included evaluation adopts a payer, health-system or societal perspective over a horizon of 15 years or longer, and none reports the position of the provider or the distribution of costs and savings between budget holders — the information an annually budgeted surgical service would need in order to act on these findings.

## Supplementary Information

Below is the link to the electronic supplementary material.

Supplementary Material 1

Supplementary Material 2

## Data Availability

All data analysed during this systematic review are derived from previously published studies, which are cited in the reference list. No new primary data were generated. The complete search log, screening log and extraction table with source references are provided as Supplementary Table S1, and the CHEERS 2022 appraisal is provided as Supplementary Table S2. Study characteristics and reported cost-effectiveness estimates are presented in the tables within the manuscript.

## Declarations

### Use of AI Tools

Claude (Anthropic) was used, with author supervision, for search-strategy translation, record parsing and de-duplication, structured data extraction and manuscript drafting, as set out in Sect.  2.5. All eligibility decisions, all verification of platform translations and extracted values against source documents, and final approval of the manuscript were performed by the author. No AI tool is credited as an author.
