# Supplementary material for: Cost-effectiveness of iStent-based trabecular micro-bypass stenting combined with cataract surgery in mild-to-moderate open-angle glaucoma: a systematic review of analytic perspectives and time horizons
Source: Cost Eff Resour Alloc. 2026 Oct 20;24:128. doi: 10.1186/s12962-026-00846-6 (PMC13685196; doi:10.1186/s12962-026-00846-6)
Supplement: Supplementary file 1 [file 12962_2026_846_MOESM1_ESM.docx]

Supplementary Table S1

**Search strategies, study selection log, excluded full-text reports and data extraction table**

*Cost-Effectiveness of iStent-Based Trabecular Micro-Bypass Stenting Combined with Cataract Surgery in Mild-to-Moderate Open-Angle Glaucoma: A Systematic Review of Analytic Perspectives and Time Horizons*

Dan Drobin, MD, PhD. Searches run 27 July 2026; screening, data extraction and appraisal completed 29 July 2026.

# S1.1 Search strategies

No date, language or publication-type restriction was applied at the search stage. Eligibility with respect to indication, model type and outcome measure was assessed at screening.

**PubMed**

Platform: NCBI E-utilities (esearch/efetch)

Search date: 27 July 2026	Records retrieved: 68

Device block: (("iStent"[tiab] OR "i-stent"[tiab] OR "trabecular micro-bypass"[tiab] OR "trabecular microbypass"[tiab] OR "micro-bypass stent"[tiab] OR "microbypass stent"[tiab] OR "trabecular bypass"[tiab] OR ("MIGS"[tiab] AND "glaucoma"[tiab]) OR "minimally invasive glaucoma surgery"[tiab] OR "minimally invasive glaucoma surgeries"[tiab] OR "micro-invasive glaucoma surgery"[tiab] OR "microinvasive glaucoma surgery"[tiab] OR "Glaucoma Drainage Implants"[Mesh]))

AND

Economics block: (("cost-effectiveness"[tiab] OR "cost effectiveness"[tiab] OR "cost-utility"[tiab] OR "cost utility"[tiab] OR "cost-benefit"[tiab] OR "cost-minimi*"[tiab] OR "economic evaluation"[tiab] OR "economic analysis"[tiab] OR "economic model*"[tiab] OR "QALY"[tiab] OR "QALYs"[tiab] OR "quality-adjusted"[tiab] OR "incremental cost"[tiab] OR "ICER"[tiab] OR "Markov"[tiab] OR "budget impact"[tiab] OR "Cost-Benefit Analysis"[Mesh] OR "Quality-Adjusted Life Years"[Mesh] OR "Economics, Medical"[Mesh] OR "Costs and Cost Analysis"[Mesh]))

**Embase**

Platform: Elsevier (embase.com), institutional access; Emtree mapping disabled

Search date: 27 July 2026	Records retrieved: 275

Device block: ('istent':ti,ab,kw OR 'i-stent':ti,ab,kw OR 'trabecular micro-bypass':ti,ab,kw OR 'trabecular microbypass':ti,ab,kw OR 'micro-bypass stent':ti,ab,kw OR 'microbypass stent':ti,ab,kw OR 'trabecular bypass':ti,ab,kw OR ('migs':ti,ab,kw AND 'glaucoma':ti,ab,kw) OR 'minimally invasive glaucoma surgery':ti,ab,kw OR 'micro-invasive glaucoma surgery':ti,ab,kw OR 'microinvasive glaucoma surgery':ti,ab,kw OR 'glaucoma drainage implant'/exp)

AND

Economics block: ('cost effectiveness analysis'/exp OR 'cost utility analysis'/exp OR 'cost benefit analysis'/exp OR 'economic evaluation'/exp OR 'quality adjusted life year'/exp OR 'health economics'/exp OR 'cost-effectiveness':ti,ab,kw OR 'cost effectiveness':ti,ab,kw OR 'cost-utility':ti,ab,kw OR 'cost-benefit':ti,ab,kw OR ('cost' NEXT/1 'minimi*'):ti,ab,kw OR 'economic evaluation':ti,ab,kw OR 'economic analysis':ti,ab,kw OR ('economic' NEXT/1 'model*'):ti,ab,kw OR 'qaly':ti,ab,kw OR 'qalys':ti,ab,kw OR 'quality-adjusted':ti,ab,kw OR 'incremental cost':ti,ab,kw OR 'icer':ti,ab,kw OR 'markov':ti,ab,kw OR 'budget impact':ti,ab,kw)

Line counts: device block 6,918; economics block 1,337,305; combination 275.

**Cochrane Library**

Platform: Wiley, Search Manager. CDSR 0; CENTRAL 16; Clinical Answers 0; Protocols 0; Editorials 0; Special Collections 0. CENTRAL Issue 6 of 12, June 2026

Search date: 27 July 2026	Records retrieved: 16

Device block: ("iStent" OR "i-stent" OR "trabecular micro-bypass" OR "trabecular microbypass" OR "micro-bypass stent" OR "microbypass stent" OR "trabecular bypass" OR "minimally invasive glaucoma surgery" OR "micro-invasive glaucoma surgery" OR "microinvasive glaucoma surgery"):ti,ab,kw OR ("MIGS":ti,ab,kw AND "glaucoma":ti,ab,kw) OR [mh "Glaucoma Drainage Implants"]

AND

Economics block: ("cost-effectiveness" OR "cost effectiveness" OR "cost-utility" OR "cost utility" OR "cost-benefit" OR (cost NEXT minimi*) OR "economic evaluation" OR "economic analysis" OR (economic NEXT model*) OR "QALY" OR "QALYs" OR "quality-adjusted" OR "incremental cost" OR "ICER" OR "Markov" OR "budget impact"):ti,ab,kw OR [mh "Cost-Benefit Analysis"] OR [mh "Quality-Adjusted Life Years"]

Line counts: device block 311; economics block 41,880; combination 16.

**Web of Science**

Platform: Clarivate, Core Collection, all editions, institutional access

Search date: 27 July 2026	Records retrieved: 59

TS=("iStent" OR "i-stent" OR "trabecular micro-bypass" OR "trabecular microbypass" OR "micro-bypass stent" OR "microbypass stent" OR "trabecular bypass" OR "minimally invasive glaucoma surgery" OR "micro-invasive glaucoma surgery" OR "microinvasive glaucoma surgery" OR "glaucoma drainage implant*" OR ("MIGS" AND "glaucoma"))

AND

TS=("cost-effectiveness" OR "cost effectiveness" OR "cost-utility" OR "cost utility" OR "cost-benefit" OR "cost minimi*" OR "economic evaluation" OR "economic analysis" OR "economic model*" OR "QALY" OR "QALYs" OR "quality-adjusted" OR "incremental cost" OR "ICER" OR "Markov" OR "budget impact")

Line counts: device block 2,123; economics block 441,978; combination 59. Run as a single combined query rather than by set number.

# S1.2 Documented syntax deviations between platforms

The device and economics blocks were held semantically constant across the four databases. Where platform syntax required a change, the change and its verification are recorded below.

| **Issue** | **Resolution and verification** |
| --- | --- |
| Truncation inside a quoted phrase | Supported in PubMed and Web of Science. The Cochrane Library returns a warning and does not execute it. The two affected terms were rewritten using the NEXT operator as recommended by Wiley: (cost NEXT minimi*) and (economic NEXT model*). Control searches confirmed no loss of recall: (cost NEXT minimi*) returned 477 records against 471 for the explicit phrase list, and (economic NEXT model*) returned 480 against 479. |
| Hyphenation inside a quoted phrase | A control search in the Cochrane Library returned 30,489 records for both ("cost-effectiveness"):ti,ab,kw and (cost NEXT effectiveness):ti,ab,kw, confirming that Wiley treats a hyphen in a quoted phrase as an adjacency operator. No adjustment was required. |
| Controlled vocabulary | PubMed (MeSH) and Embase (Emtree) support controlled-vocabulary terms; Web of Science does not. The Web of Science block is therefore free-text only, and TS= covers title, abstract, author keywords and KeyWords Plus. |
| Qualification of the acronym MIGS | "MIGS" is ambiguous and also denotes minimally invasive gynaecologic surgery. It was qualified against "glaucoma" in all four databases. In PubMed this removed six records, all non-ophthalmic, and no relevant record. |
| Eligibility of health technology assessments | Health technology assessment reports were excluded as a publication type, which is the criterion applied in the submitted version of this review. Two such reports were retrieved by the new search and both are listed among the full-text exclusions in S1.4. |
| Eligible model types | The criterion specified for this review is a formal decision-analytic model. Both cohort Markov models and individual-patient microsimulations are decision-analytic models and both are eligible. Model type is reported as a study characteristic in Table 1 and as a source of heterogeneity in Section 4.1. |
| De-duplication | Records were matched on DOI (96 pairs), PubMed identifier (3) and normalised title with year (23). Five further candidate pairs above a fuzzy-matching threshold of 92 were reviewed individually: three were the same ISPOR abstract indexed in both Embase and Web of Science, where Embase retains the session code in the title field and the Web of Science export carries no DOI, and were merged; two were a letter and its published response, with separate DOIs and different first authors, and were retained as distinct records. |

# S1.3 Study selection

| **Stage** | **n** |
| --- | --- |
| Records identified from databases | 418 |
| — PubMed | 68 |
| — Embase | 275 |
| — Cochrane CENTRAL | 16 |
| — Web of Science | 59 |
| Duplicate records removed | 125 |
| Records screened | 293 |
| Records excluded on publication type | 98 |
| — conference abstracts | 51 |
| — correspondence | 18 |
| — editorials | 14 |
| — registry entries | 9 |
| — other publication types | 6 |
| Reviews identified but not eligible as primary studies | 17 |
| Records screened on title and abstract | 178 |
| Records excluded on title and abstract | 155 |
| Reviews identified but not eligible as primary studies, this stage | 3 |
| Reports sought for retrieval | 20 |
| Reports not retrieved | 2 |
| Reports assessed for eligibility | 18 |
| Reports excluded at full text | 10 |
| Studies included in the review | 8 |

Screening, full-text assessment, data extraction and appraisal were performed by a single reviewer (DD). No independent duplicate screening was undertaken. Every extracted value carries a page or section reference to its source, given in S1.5.

# S1.4 Reports excluded at full text, with reasons

| **Report** | **Title** | **Reason for exclusion** | **Basis** |
| --- | --- | --- | --- |
| Gravina 2023 | Cost-utility of primary non-pharmacological treatment of open-angle glaucoma | not combined with cataract surgery | iStent inject implanted after failure of selective laser trabeculoplasty; no cataract surgery in any arm (p. 1) |
| Guedes 2022 | A Brazilian cost-utility analysis of trabecular micro-bypass with iStent inject® for the treatment of open-angle glaucoma | not combined with cataract surgery | iStent inject versus topical medication as a standalone procedure; cataract surgery appears only in the reference list (p. 1) |
| Ordonez 2019 | Cost-effectiveness analysis of iStent trabecular micro-bypass stent for patients with open-angle glaucoma in Colombia. | not combined with cataract surgery | Standalone stent versus selective laser trabeculoplasty and medication regimens (pp. 3, 5, 7) |
| Patel 2019 | Cost-effectiveness analysis of standalone trabecular micro-bypass stents in patients with mild-to-moderate open-angle glaucoma in Canada. | not combined with cataract surgery | "Standalone trabecular micro-bypass stents" per the title; separate procedure versus medication alone |
| Ontario Health (Quality) 2021 | iStent for Adults With Glaucoma: A Health Technology Assessment. | not a primary peer-reviewed economic evaluation | Health technology assessment report, not a primary peer-reviewed economic evaluation. It additionally contains no original economic model: its economic section, headed "Included Economic Evaluations" and "Additional Economic Evaluations", summarises four published analyses (CADTH ref 29; INESSS ref 23; Ahmed 2020 ref 36; Patel 2019 ref 37). Inclusion would have double-counted Ahmed 2020, which is included in its own right |
| Poitras 2019 | Optimal Use of Minimally Invasive Glaucoma Surgery: A Health Technology Assessment | not a primary peer-reviewed economic evaluation | Health technology assessment report. Contains an original economic model (Model 4) but is not a primary peer-reviewed economic evaluation, which is the publication-type criterion applied throughout this review |
| Guedes 2021 | Preventing Glaucoma Progression using the Trabecular Micro-Bypass Implant IStent Inject®. A Cost-Effectiveness Analysis | outcome not QALY | Progression-free life-years as the effect measure; willingness to pay expressed per PFLY (pp. 1, 4) |
| Tan 2016 | Manchester iStent study: 3-year results and cost analysis. | outcome not QALY | Interventional case series with cost analysis; no Markov model and no QALY (pp. 1–2) |
| Ontario Health (Quality) 2019 | Minimally Invasive Glaucoma Surgery: A Budget Impact Analysis and Evaluation of Patients' Experiences, Preferences, and Values. | device not iStent | Budget impact calculated for MIGS as a class in three subgroups; iStent pooled with Hydrus, CyPass and endoscopic cyclophotocoagulation, with no iStent-specific estimate reported |
| Li 2023 | Cost-utility analysis of commonly used anti-glaucoma interventions for mild-to-moderate primary open-angle glaucoma patients in rural and urban China | wrong intervention | Models maximal medical treatment, trabeculectomy, Ahmed valve, GATT and ab interno canaloplasty; no iStent in the model (p. 1) |

Two further reports were sought but could not be obtained by any route and remain unassessed: Berdahl JP et al., Cost-comparison of two trabecular micro-bypass stents versus selective laser trabeculoplasty or medications only for intraocular pressure control for patients with open-angle glaucoma; and Iordanous Y et al., Projected cost comparison of Trabectome, iStent, and endoscopic cyclophotocoagulation versus glaucoma medication in the Ontario Health Insurance Plan. One included study (Sood et al. 2022) was assessed from the published abstract and the publisher's article preview because the author's institution does not hold a subscription to the journal.

# S1.5 Data extraction table

Data extraction was AI-assisted, as described in Section 2.5 of the manuscript. Every value carries a page or section reference to its source and was checked against the source document by the author. NR indicates that the item is not reported in the source; where a value could not be verified because the full text was unavailable, this is stated.

**Ahmed 2020**

| **Item** | **Extracted value and source reference** |
| --- | --- |
| Country | Canada [abstract] |
| Population | Modelled cohort with open-angle glaucoma and visually significant cataract; 83.1% mild, 16.9% moderate [abstract] |
| Intervention | iStent inject TBS + cataract surgery [abstract] |
| Device modelled | iStent inject (two stents) [abstract] |
| Comparator | Cataract surgery alone [abstract] |
| Combined / standalone | Combined [abstract] |
| Model type | Markov, Hodapp-Parrish-Anderson stages (mild 0 to −6 dB; moderate −6.01 to −12; advanced −12.01 to −20; severe/blind below −20) plus death. Monthly cycles [p. 104] |
| Perspective | Canadian public payer [abstract] |
| Time horizon | 15 years [p. 104] |
| Discount rate, costs | 1.5% per CADTH guidelines; 0% and 3% in scenario analyses [p. 105] |
| Discount rate, outcomes | 1.5% [p. 105] |
| Currency | CAD |
| Price year | 2017 [p. 105, Table 1] |
| Result | ICUR −CAD 16,691; the TBS arm dominates in the base case. 95% of simulations below CAD 62,366 per QALY. Net cost −CAD 389.00 (95% CI −1,712.00 to +850.70); +0.023 QALY (95% CI 0.004–0.044) [Table 3, p. 108] |
| Unit | CAD per QALY (dominant in the base case) |
| Threshold applied | CAD 50,000 and CAD 100,000 per QALY [p. 108] |
| Probability at threshold | 94.1% at CAD 50,000 per QALY; 97.0% at CAD 100,000. 73.7% probability of cost saving; 98.8% probability of greater effectiveness [p. 108] |
| Funding | Glaukos Corporation, San Clemente; grant to Pharmerit International LP to develop the model and the manuscript. The sponsor participated in preparation, review and approval of the manuscript [p. 113] |
| Conflicts of interest | Falvey and Murray are employees of Glaukos Corporation. Patel and Botteman were employed by Pharmerit International, funded by Glaukos. Goeree serves on a Glaukos advisory board. Ahmed is a consultant for and has received research support from Glaukos among some forty other companies. Podbielski is a consultant for Glaukos [pp. 112–113] |
| Notes | Scenario analyses, Table 4, p. 110 — the same model over three horizons: lifetime, TBS dominates, 95th percentile CAD 55,952; 15 years (base case), TBS dominates, ICUR −CAD 16,691; five years, TBS does NOT dominate, ICUR CAD 32,227.80 per QALY with an incremental cost of +CAD 446.90 and a 95th percentile of CAD 125,941. Discounting at 0% and at 3%: dominant. Societal perspective: dominant, 95th percentile CAD 86,638. Medication discontinuation without IOP benefit: ICUR CAD 19,212.50. Cost drivers, Table 3: primary surgery +CAD 1,671.50; medication −CAD 2,069.60 — the whole net saving lies in reduced medication use |

**Bartelt-Hofer 2020**

| **Item** | **Extracted value and source reference** |
| --- | --- |
| Country | Germany [p. 1] |
| Population | Primary open-angle glaucoma, stratified by stage; the moderate-stage arms form the synthesis arm [p. 1] |
| Intervention | iStent, one and two stents, implanted with cataract surgery [p. 1] |
| Device modelled | Trabecular micro-bypass stent (TMBS), one and two stents in separate arms. NOTE: in Table 7 the abbreviation IS denotes intracanalicular scaffold (Hydrus), not iStent; see the table footnote on p. 4 |
| Comparator | Cataract surgery alone [Table 7, p. 8] |
| Combined / standalone | Combined [p. 1] |
| Model type | Markov disease-transition model across POAG stages, yearly cycles [pp. 2, 5] |
| Perspective | German payer [p. 1] |
| Time horizon | Lifetime, yearly cycles [p. 5] |
| Discount rate, costs | 3% [p. 5] |
| Discount rate, outcomes | 3% [p. 5] |
| Currency | EUR |
| Price year | 2019 [p. 5] |
| Result | Table 7, p. 8 — all rows versus CATARACT SURGERY ALONE. Moderate POAG: one TMBS + cataract EUR 15,673.41 per QALY (incremental cost EUR 76.55); two TMBS + cataract EUR 10,955.20 per QALY (incremental cost EUR 137.25). Advanced POAG: one TMBS EUR 86,942.73 per QALY; two TMBS EUR 60,269.14 per QALY. Two TMBS in moderate disease is both the most effective strategy and the one with the lowest cost per QALY gained |
| Unit | EUR per QALY |
| Threshold applied | One times GDP per capita, EUR 43,433.87 [p. 8] |
| Probability at threshold | Over 95% of simulations fall below EUR 43,433.87 [p. 8] |
| Funding | No specific funding; open access via Projekt DEAL [p. 9] |
| Conflicts of interest | The authors declare no conflict of interest [p. 9] |
| Notes | Monte Carlo appears only as the probabilistic sensitivity analysis method, not as the model class. The model compares four MIGS approaches but iStent is modelled in two separate arms. The IS arm (intracanalicular scaffold, EUR 82,001.22 per QALY in moderate disease) is not part of the synthesis |

**Fea 2021**

| **Item** | **Extracted value and source reference** |
| --- | --- |
| Country | Italy [p. 1] |
| Population | Mild-to-moderate open-angle glaucoma [p. 1] |
| Intervention | iStent inject + concurrent cataract surgery [p. 1] |
| Device modelled | iStent inject [p. 1] |
| Comparator | Cataract surgery alone [p. 1] |
| Combined / standalone | Combined [p. 1] |
| Model type | Markov, four health states, one-month cycles [p. 1] |
| Perspective | Italian National Health Service; direct healthcare costs only [p. 1] |
| Time horizon | Lifetime [p. 2] |
| Discount rate, costs | 3.5% |
| Discount rate, outcomes | 3.5% |
| Currency | EUR |
| Price year | NR — no price year is stated. Costs are drawn from regional and national tariff decisions of mixed vintage (DGR 2011, 2013 and 2016; Gazzetta Ufficiale 2013), all accessed 1 July 2021, with no common price year and no inflation adjustment reported [p. 6; reference list p. 11] |
| Result | ICER EUR 13,037.01 per QALY [p. 1]. Lifetime total cost EUR 8,368.51 versus EUR 7,134.71 |
| Unit | EUR per QALY |
| Threshold applied | EUR 50,000 per QALY [p. 1] |
| Probability at threshold | 98% at EUR 50,000 per QALY [p. 1; CEAC Figure 3, p. 8] |
| Funding | Glaukos [p. 10] |
| Conflicts of interest | Buseghin G is affiliated with Glaukos [author affiliations, p. 1]. Furneri G: consulting fees from Glaukos for conducting the analysis. Fea AM: consulting fees from Glaukos, Ivantis, ELT Sight, iSTAR and EyeD [p. 10] |
| Notes | — |

**Nieland 2021**

| **Item** | **Extracted value and source reference** |
| --- | --- |
| Country | France [p. 1] |
| Population | Mild-to-moderate open-angle glaucoma [p. 1] |
| Intervention | iStent inject + phacoemulsification cataract surgery [p. 1] |
| Device modelled | iStent inject [p. 1] |
| Comparator | Cataract surgery alone [p. 1] |
| Combined / standalone | Combined [p. 1] |
| Model type | Markov, adapted from a previously published model in accordance with French health-economic guidelines [p. 3]; cycle length one month [Table 1, p. 4] |
| Perspective | Societal [p. 3; Table 1, p. 4] |
| Time horizon | Lifetime [p. 5] |
| Discount rate, costs | 4% [p. 5] |
| Discount rate, outcomes | 4% [p. 5] |
| Currency | EUR |
| Price year | 2019 — French tariffs are reported in 2019 Euros and costs extracted from the literature were inflated to 2019 values using the French consumer price index [p. 5] |
| Result | ICER EUR 1,154 per QALY [p. 7] |
| Unit | EUR per QALY |
| Threshold applied | EUR 30,000 per QALY [p. 5] |
| Probability at threshold | 86% at a willingness to pay of EUR 30,000 per QALY [p. 2, abstract; p. 9, discussion] |
| Funding | Glaukos Corporation, paid to Pharmerit International; two co-authors (Falvey H, Gicquel G) are employees of the manufacturer [funding statement] |
| Conflicts of interest | Falvey H and Gicquel G are employees and stockholders of Glaukos; Nieland K received consulting fees from Glaukos; the Pharmerit authors are current or former employees of the funded consultancy [competing interests statement] |
| Notes | — |

**Teus 2021**

| **Item** | **Extracted value and source reference** |
| --- | --- |
| Country | Spain [p. 1] |
| Population | Mild-to-moderate primary open-angle glaucoma [p. 1] |
| Intervention | iStent inject implanted during cataract surgery [p. 1] |
| Device modelled | iStent inject [p. 1] |
| Comparator | Cataract surgery alone [p. 1] |
| Combined / standalone | Combined [p. 1] |
| Model type | Markov health-state transition model, cycle length one month [p. 3] |
| Perspective | Spanish health system [p. 2] |
| Time horizon | Lifetime, taking 100 years as maximum life expectancy in Spain; monthly cycles [p. 3, section 2.2]. NOTE: the results table on p. 6 carries the footnote “*Time horizon 10 years”. That footnote is mislabelled in the source: p. 5 states that treatment efficacy was extrapolated to 10 years, after which the intraocular pressure reduction is assumed negligible and the model continues to end of life following the natural course of the disease (confirmed p. 9). Ten years is the efficacy horizon, not the analytic horizon. The accumulated 12.36 and 12.27 QALYs are not attainable over 10 years and confirm the lifetime horizon |
| Discount rate, costs | Discounting applied per Spanish guidelines; numerical rate not stated in the text [p. 3] |
| Discount rate, outcomes | As above [p. 3] |
| Currency | EUR |
| Price year | 2020 — Costs (EUR, 2020) were obtained from the Spanish database eSalud [p. 2]; Table 2 p. 5 and the cost table p. 6 both use the column heading Unit cost (EUR, 2020) |
| Result | ICER EUR 12,676.48 per QALY, iStent inject + cataract surgery versus cataract surgery alone. Total cost EUR 13,344.05 versus EUR 12,293.86; the difference column of the same table reports EUR 1,002.07, whereas the two totals differ by EUR 1,050.19 and the reported ICER is consistent with the latter. 12.36 versus 12.27 QALYs, incremental effect 0.083 QALY [table p. 6; pp. 2, 8, 9] |
| Unit | EUR per QALY |
| Threshold applied | EUR 21,000 per QALY (Spanish threshold) and EUR 30,000 per QALY (conventional) [p. 7] |
| Probability at threshold | 90.9% at EUR 30,000 per QALY; 78.4% at EUR 21,000 per QALY [pp. 2, 3] |
| Funding | Glaukos Corporation [p. 10] |
| Conflicts of interest | All eight authors disclose a relationship with Glaukos. Falvey H and Buseghin G are employees of Glaukos. Teus MA, Belda JI, Lavín C, García-Feijoó J, Soler M and Appierto M have acted as consultants for Glaukos; Teus MA also for or with research support from Alcon, Allergan, Johnson & Johnson, Novartis, Santen and Thea [Declaration of interest, p. 10] |
| Notes | Scenario analyses reported on p. 6: societal perspective, 15-year horizon, and 100% persistence. Outcome on p. 9: over a 15-year horizon the ICER was EUR 15,699.56, against EUR 12,676.48 in the lifetime base case. This is a second instance of the same model reported over two horizons, alongside Ahmed et al. |

**Healey 2022**

| **Item** | **Extracted value and source reference** |
| --- | --- |
| Country | Australia [p. 344] |
| Population | Open-angle glaucoma; the synthesis arm is the population undergoing cataract surgery [p. 344] |
| Intervention | Trabecular bypass device (iStent and iStent inject, pooled as a single device class) implanted with cataract surgery [p. 344] |
| Device modelled | iStent and iStent inject pooled. The two generations are not modelled in separate arms |
| Comparator | Usual care: cataract surgery alone with continued topical medication [p. 346] |
| Combined / standalone | Both populations modelled separately. SYNTHESIS ARM: the population undergoing cataract surgery. The standalone population (continued medical management as comparator) is reported but not used here |
| Model type | Individual-patient Monte Carlo microsimulation in TreeAge, 500,000 simulated patients [section 2.8] |
| Perspective | Australian health care system, third-party payer. Indirect costs and patient out-of-pocket costs excluded [p. 346] |
| Time horizon | 15 years, deliberately limited to avoid extrapolating beyond the available evidence [p. 346] |
| Discount rate, costs | 5%, per MSAC guidelines [p. 346] |
| Discount rate, outcomes | 5% [p. 346] |
| Currency | AUD |
| Price year | 2017 [p. 346] |
| Result | SYNTHESIS ARM, cataract surgery population: ICER A$2,430 per QALY; incremental cost A$177; incremental effect 0.0726 QALY. Standalone population, not in the synthesis: ICER A$14,644 per QALY; incremental cost A$2,234; 0.1526 QALY |
| Unit | AUD per QALY |
| Threshold applied | A$50,000 per QALY (conventional Australian reference range) [p. 353] |
| Probability at threshold | NR — no probabilistic sensitivity analysis was performed. The authors state that the computational burden of the microsimulation precluded it, and report deterministic sensitivity analyses instead [section 2.8; discussion] |
| Funding | Glaukos Corporation; THEMA Consulting received consulting fees for the analysis. Prepared as supporting evidence for an MSAC application [p. 355] |
| Conflicts of interest | Tilden is a director of THEMA Consulting; two further authors are THEMA employees. Healey has received honoraria from Glaukos and Ivantis [p. 355] |
| Notes | Model class is an individual-patient microsimulation, not a cohort Markov model. This meets the eligibility criterion specified for this review, which requires a formal decision-analytic model. The deliberate 15-year cap is a second short-horizon data point alongside Ahmed et al. and is used in Section 4.2. Study selection in that paper was performed by a single reviewer, which the authors state explicitly |

**Igarashi 2022**

| **Item** | **Extracted value and source reference** |
| --- | --- |
| Country | Japan [p. 1] |
| Population | Mild-to-moderate primary open-angle glaucoma [p. 1] |
| Intervention | iStent inject + cataract surgery, one eye [p. 1] |
| Device modelled | iStent inject [p. 1] |
| Comparator | Cataract surgery alone [p. 1] |
| Combined / standalone | Combined [p. 1] |
| Model type | Markov health-state transition model adapted from a Canadian model, monthly cycle length [p. 2] |
| Perspective | Japanese public payer [p. 1] |
| Time horizon | Lifetime, monthly cycles. The study reports ONE horizon only — there is no five-year analysis and no other horizon scenario [pp. 1, 2, 5] |
| Discount rate, costs | 2.0% per year, per the Japanese guideline [p. 2] |
| Discount rate, outcomes | 2.0% per year [p. 2] |
| Currency | JPY |
| Price year | April 2021 — “All costs were as of April 2021”; medication and adverse-event costs from the MHLW, April 2021 [Costs section, p. 3; Table 1] |
| Result | ICUR JPY 1,430,647 per QALY — the probabilistic BASE CASE over a LIFETIME horizon, iStent inject + cataract surgery versus cataract surgery alone. Total cost JPY 1,025,785 versus JPY 933,759 (incremental JPY 92,026); 12.80 versus 12.74 QALYs (incremental 0.06); 16.68 versus 16.66 life-years. Secondary outcome: ICER JPY 12,845,154 per blind eye avoided [pp. 1, 5; Table 2, p. 5] |
| Unit | JPY per QALY |
| Threshold applied | JPY 5,000,000 per QALY [p. 4] |
| Probability at threshold | 90% at JPY 5,000,000 per QALY [p. 5] |
| Funding | Glaukos Corporation; analysis and medical writing by Creativ-Ceutical [p. 7] |
| Conflicts of interest | Chu A and Falvey H are employees and stockholders of Glaukos; Han R, Ueyama M and Onishi Y are employees of Creativ-Ceutical. Igarashi A, Ishida K and Shoji N received consulting fees for study design, interpretation of results and review of the manuscript [p. 7] |
| Notes | Scenario analysis from a societal perspective including caregiver burden: iStent inject dominates. The full text was searched for the terms “five-year” and “5-year” with no result; the study models a single lifetime horizon, and JPY 1,430,647 is the base-case ICUR over that horizon |

**Sood 2022**

| **Item** | **Extracted value and source reference** |
| --- | --- |
| Country | United States [abstract] |
| Population | Adults aged 65 and over with mild-to-moderate primary open-angle glaucoma, with or without cataract [abstract] |
| Intervention | Trabecular meshwork bypass stent implanted at the time of cataract surgery [Study Design, article preview] |
| Device modelled | iStent inject in its own arm; Hydrus Microstent in a separate arm [Study Design, article preview] |
| Comparator | Cataract surgery alone; secondarily Hydrus Microstent and medication management [abstract] |
| Combined / standalone | Combined [Study Design, article preview] |
| Model type | Markov, four glaucoma states (mild, moderate, advanced, severe/blind) plus death [Study Design, article preview] |
| Perspective | Societal, United States; costs at Medicare rates [abstract] |
| Time horizon | Lifetime, operationalised as 35 years [Study Design, article preview] |
| Discount rate, costs | NR — full text required |
| Discount rate, outcomes | NR — full text required |
| Currency | USD |
| Price year | NR — the abstract states Medicare rates but no price year |
| Result | Dominant. iStent inject + cataract surgery USD 49,599.86 and 12.21 QALYs versus cataract surgery alone USD 54,409.25 and 12.04 QALYs: USD 4,809.39 less and 0.17 QALYs more. Medication-only reference arm USD 57,931.22 and 11.74 QALYs. Hydrus arm, not in the synthesis: USD 48,026.13 and 12.26 QALYs. The device arms dominated or were cost-effective versus cataract surgery alone within five years [abstract] |
| Unit | Dominant (cost saving) |
| Threshold applied | USD 50,000 per QALY [abstract] |
| Probability at threshold | 94.19% of iterations [abstract] |
| Funding | Research to Prevent Blindness. No additional study-specific funding was received beyond the authors' regular institutional appointments [funding statement, article preview] |
| Conflicts of interest | No proprietary or commercial interest declared [disclosure statement, article preview] |
| Notes | Full text not available: the author's institution does not hold a subscription to Ophthalmology Glaucoma (Elsevier). Data are taken from the publisher's article preview: the abstract plus section snippets from Study Design, Model Validation and Discussion. Model class and time horizon are therefore source-verified rather than assumed. The preview also reports model validation against the Malmo and Olmsted studies: after 20 years, 21.14% had progressed to severe glaucoma or unilateral blindness. Eight CHEERS items could not be assessed; funding and conflicts of interest are stated in the preview and were assessed |
