# Supplementary material for: Cost-effectiveness of iStent-based trabecular micro-bypass stenting combined with cataract surgery in mild-to-moderate open-angle glaucoma: a systematic review of analytic perspectives and time horizons
Source: Cost Eff Resour Alloc. 2026 Oct 20;24:128. doi: 10.1186/s12962-026-00846-6 (PMC13685196; doi:10.1186/s12962-026-00846-6)
Supplement: Supplementary file 2 [file 12962_2026_846_MOESM2_ESM.docx]

Supplementary Table S2

**Item-by-item appraisal of the included studies against CHEERS 2022**

CHEERS 2022 is applied here as an assessment of reporting completeness, not as a risk-of-bias or quality instrument. The developers state explicitly that the checklist is not intended as a quality-assessment tool. Each item asks whether a given piece of information is reported in the publication, not whether the study is well conducted. The appraisal is therefore a factual check with reproducible answers, which is the basis on which it was undertaken by a single reviewer (DD, 29 July 2026).

Item numbering follows Husereau et al., BMJ 2022;376:e067975. Sood et al. 2022 could not be obtained in full text; items requiring inspection of the methods are marked "unable to assess" rather than "no", since absence of verifiability is not absence of reporting.

| **#** | **CHEERS 2022 item** | **Ahmed 2020** | **Bartelt-Hofer 2020** | **Fea 2021** | **Igarashi 2022** | **Nieland 2021** | **Teus 2021** | **Sood 2022** | **Healey 2022** |
| --- | --- | --- | --- | --- | --- | --- | --- | --- | --- |
| 1 | Title | Yes | Yes | Yes | Yes | Yes | Yes | Yes | Yes |
| 2 | Abstract | Yes | Yes | Yes | Yes | Yes | Yes | Yes | Yes |
| 3 | Background and objectives | Yes | Yes | Yes | Yes | Yes | Yes | Yes | Yes |
| 4 | Health economic analysis plan | No | No | No | No | No | No | No | No |
| 5 | Study population | Yes | Yes | Yes | Yes | Yes | Yes | Yes | Yes |
| 6 | Setting and location | Yes | Yes | Yes | Yes | Yes | Yes | Yes | Yes |
| 7 | Comparators | Yes | Yes | Yes | Yes | Yes | Yes | Yes | Yes |
| 8 | Perspective | Yes | Yes | Yes | Yes | Yes | Yes | Yes | Yes |
| 9 | Time horizon | Yes | Yes | Yes | Yes | Yes | Yes | Yes | Yes |
| 10 | Discount rate | Yes | Yes | Yes | Yes | Yes | No | n/a | Yes |
| 11 | Selection of outcomes | Yes | Yes | Yes | Yes | Yes | Yes | Yes | Yes |
| 12 | Measurement of outcomes | Yes | Yes | Yes | Yes | Yes | Yes | n/a | Yes |
| 13 | Valuation of outcomes | Yes | Yes | Yes | Yes | Yes | Yes | n/a | Yes |
| 14 | Measurement and valuation of resources and costs | Yes | Yes | Yes | Yes | Yes | Yes | n/a | Yes |
| 15 | Currency, price date, and conversion | Yes | Yes | No | Yes | Yes | Yes | n/a | Yes |
| 16 | Rationale and description of model | Yes | Yes | Yes | Yes | Yes | Yes | Yes | Yes |
| 17 | Analytics and assumptions | Yes | Yes | Yes | Yes | Yes | Yes | n/a | Yes |
| 18 | Characterising heterogeneity | No | Yes | No | No | Yes | No | n/a | No |
| 19 | Characterising distributional effects | No | No | No | No | No | No | No | No |
| 20 | Characterising uncertainty | Yes | Yes | Yes | Yes | Yes | Yes | Yes | Yes |
| 21 | Approach to engagement with patients | No | No | No | No | No | No | No | No |
| 22 | Study parameters | Yes | Yes | Yes | Yes | Yes | Yes | n/a | Yes |
| 23 | Summary of main results | Yes | Yes | Yes | Yes | Yes | Yes | Yes | Yes |
| 24 | Effect of uncertainty | Yes | Yes | Yes | Yes | Yes | Yes | Yes | Yes |
| 25 | Effect of engagement with patients | No | No | No | No | No | No | No | No |
| 26 | Findings, limitations, generalisability | Yes | Yes | Yes | Yes | Yes | Yes | Yes | Yes |
| 27 | Source of funding | Yes | Yes | Yes | Yes | Yes | Yes | Yes | Yes |
| 28 | Conflicts of interest | Yes | Yes | Yes | Yes | Yes | Yes | Yes | Yes |

Yes = the item is reported. No = the item is not reported. n/a = unable to assess because the full text was not available.

**Summary of recurring shortcomings**

No study reported a pre-specified health economic analysis plan (item 4), characterised distributional effects (item 19), or reported patient or stakeholder engagement (items 21 and 25). Heterogeneity between patient groups (item 18) was characterised by two studies: by disease stage in Bartelt-Hofer and Flessa, and by degree of medication persistence in Nieland et al. The price year (item 15) was reported by six studies, spanning 2017 to 2021, was not reported by one, and could not be verified for one. Reporting was substantially complete across the remaining items in all eight studies. Healey et al. 2022 was appraised on 29 July 2026 against the full text.
